# Cytoplasmic SIRT1 inhibits cell migration and invasion by impeding epithelial–mesenchymal transition in ovarian carcinoma

**DOI:** 10.1007/s11010-019-03559-y

**Published:** 2019-07-17

**Authors:** Tong Yang, Ru Zhou, Shentong Yu, Shuhong Yu, Zhuqing Cui, Peizhen Hu, Jinsong Liu, Qing Qiao, Jing Zhang

**Affiliations:** 1 State Key Laboratory of Cancer Biology, Department of Pathology, Xijing Hospital, The Fourth Military Medical University, No. 169, Changle West Road, Xi’an, 710032 Shaanxi China; 2 Department of Pathology, The University of Texas M.D. Anderson Cancer Center, Houston, TX 77030 USA; 3 Department of General Surgery, Tangdu Hospital, The Fourth Military Medical University, No. 569 Xinsi Road, Xi’an, 710038 Shaanxi China

**Keywords:** Sirtuin1, Subcellular localization, Carcinoma, Ovarian epithelial, Cell movement, Neoplasm invasiveness, Epithelial–mesenchymal transition

## Abstract

Sirtuin1 (SIRT1) is a mammalian NAD^+^-dependent type III deacetylase that plays paramount roles in diverse cellular processes. The nucleocytoplasmic shuttling of SIRT1 was discovered more than a decade ago, but the roles of subcellular SIRT1 localization in tumor progression remain unclear. Here, we report that cytoplasmic SIRT1 acts as a tumor suppressor in ovarian carcinoma. By creating ovarian carcinoma cell lines overexpressing wild-type SIRT1 and nuclear localization signals (NLSs) mutated SIRT1 together with both unbiased proteomic and acetylomic approaches and Transwell assays, we identified that mutations in the NLS sequences prevented SIRT1 from entering the nucleus, resulting in the predominant cytoplasmic localization of SIRT1; the cytoplasmic localization of SIRT1 suppressed the mesenchymal program, activated the epithelial program, and inhibited the migration and invasion of tumor cells, thus providing experimental evidence that SIRT1 functions as a tumor suppressor or oncogene may depend on its subcellular localization. Altogether, our findings may highlight a novel role of cytoplasmic SIRT1 in ovarian carcinoma, providing new possible insights for studies investigating the role of SIRT1 in tumor progression.

## Introduction

Epithelial ovarian carcinoma remains the most lethal gynecologic malignancy worldwide. In 2018, 22,240 new ovarian cancer cases and 14,070 ovarian cancer deaths were projected to occur in the USA [1]. With the development of surgical techniques and adjuvant treatment with chemotherapeutic drugs, such as platinum and paclitaxel, the median survival duration of patients with ovarian carcinoma has improved, but worldwide, the survival trends have shown very minimal improvement in the 5-year survival rate [2]. Due to its occult onset, ovarian carcinoma is usually found in an advanced tumor stage with extensive abdominal implantation and/or distant metastasis, greatly contributing to its high mortality.

Sirtuin1 (SIRT1) is a mammalian nicotinamide adenine dinucleotide (NAD^+^)-dependent type III deacetylase that belongs to a conserved and versatile family of biological regulators. SIRT1 and its homologous proteins exist in almost all eukaryotic cells from yeast to humans. SIRT1 regulates many intracellular factors, such as p53 [3], FOXO1 [4], nuclear factor κB [5], and E2F1 [6], and plays paramount roles in diverse cellular processes, including oxidative stress [7], apoptosis [8], metabolism [9], cell proliferation and differentiation [10], and autophagy [11].

To date, the role of SIRT1 in tumor progression remains controversial. Accumulating evidence indicates that SIRT1 may play a biphasic role in this process [12]. On the one hand, SIRT1 maintains genomic stability, blocks the initiation of carcinogenesis by counteracting genotoxic insults [13], and participates in DNA repair processes [14]; on the other hand, SIRT1 facilitates cell survival and contributes to the acquisition of chemoresistance [15, 16]. Existing data clearly suggest that SIRT1 can shuttle between the nucleus and cytoplasm in a manner dependent on its two nuclear localization signals (NLSs) and two nuclear export signals (NESs). If the two NLSs or two NESs are simultaneously mutated, SIRT1 loses its shuttling capacity and is restrained in the cytoplasm or nucleus [17]. Therefore, a possible explanation for the aforementioned controversial issues is the subcellular localization of SIRT1 [17, 18]. However, this hypothesis has never been experimentally tested.

In the current study, we compared the differences in migration and invasion between tumor cells overexpressing wild-type SIRT1 or NLS-mutated SIRT1 and further explored the related mechanisms in vitro. Our results suggest that cytoplasmic SIRT1 inhibits cell migration and invasion by impeding the epithelial–mesenchymal transition (EMT) in ovarian carcinoma, thus providing the first experimental evidence supporting the possible role of SIRT1 subcellular localization in the regulation of ovarian carcinoma progression.

## Materials and methods

### Cell culture

The human ovarian carcinoma cell line IGROV1 was purchased from the American Type Culture Collection (ATCC, Manassas, VA, USA). The cells were cultured in MEM/EBSS medium (HyClone, Logan, UT, USA) supplemented with 1 mM sodium pyruvate, 10% heat-inactivated fetal bovine serum (FBS, Sijiqing Co., Ltd., Hangzhou, Zhejiang, China), penicillin (100 U/ml), and streptomycin (100 μg/ml) at 37 °C in a humidified atmosphere of 5% CO_2_.

### Lentivirus infection

Lentiviral vectors with the complete coding sequence of SIRT1 (lenti-SIRT1) and NLS-mutant SIRT1 (lenti-SIRT1^NLSmt^) and a control empty vector (Con136) were purchased from GeneChem (GeneChem, Shanghai, China). The sites of two NLSs were mutated according to a study conducted by Tanno et al. [17]. The exogenous proteins of SIRT1 and SIRT1^NLSmt^ were tagged with green fluorescence protein (GFP). The IGROV1 cells were transfected with Con136, lenti-SIRT1, and lenti-SIRT1^NLSmt^ and selected with 6 μg/ml puromycin for 48 h. The mRNA and protein expression levels and subcellular location of SIRT1 were confirmed by real-time PCR and Western blot analyses.

### Immunofluorescence staining

The cells were cultured in a 24-well plate and fixed in 4% paraformaldehyde for 15 min at room temperature. After treatment with 0.2% Triton X-100 in PBS for 15 min at room temperature and blocking for 30 min with 1% BSA in PBS, the cells were incubated with an anti-GFP antibody (1:100; ProteinTech, Wuhan, Hubei, China) at 4 °C overnight. Then, the cells were incubated for 30 min at room temperature with a FITC-conjugated anti-rabbit IgG antibody (1:100; CWBIO, Beijing, China) and stained with DAPI (10 μg/ml; Solarbio, Beijing, China) for 5 min at room temperature. The staining results were assessed under an inversion fluorescence microscope (Olympus BX51WI, Tokyo, Japan).

### RNA extraction and real-time PCR

RNA was extracted using RNAiso Plus (TaKaRa, Kusatsu, Shiga, Japan) according to the manufacturer’s instructions. The reverse transcription reactions were performed using PrimerScript® RT Master Mix (Perfect Real Time) (TaKaRa). Real-time PCR was carried out by using SYBR Premix Ex Taq II (TaKaRa) on an Mx3000P Real-Time PCR system (StrataGene, CA, USA). The primers used for the real-time PCR analysis are listed in Table 1. Triplicate wells were analyzed, and each experiment was repeated three times. Then, the relative quantitative values were calculated by the 2^−ΔΔCt^ method.

**Table 1:** Primers used for the real-time PCR analysis

Primers	Sequences
β-Actin forward	5′-GGACTTCGAGCAAGAGATGG-3′
β-Actin reverse	5′-AGCACTGTGTTGGCGTACAG-3′
SIRT1 forward	5′-GCAGATTAGTAGGCGGCTTG-3′
SIRT1 reverse	5′-TCATCCTCCATGGGTTCTTC-3′
Fibronectin forward	5′-CAGACAACCATCTCATGGG-3′
Fibronectin reverse	5′-AACCCTGAACTGTAAGGGT-3′
Vimentin forward	5′-GAGTCCACTGAGTACCGGAGAC-3′
Vimentin reverse	5′-TGTAGGTGGCAATCTCAATGTC-3′
CK-18 forward	5′-TTGATGACACCAATATCACACGA-3′
CK-18 reverse	5′-TATTGGGCCCGGATGTCTG-3′
CK-19 forward	5′-GCGGCCAACGGCGAGCTA-3′
CK-19 reverse	5′-GCAGGACAATCCTGGAGTTCTC-3′
Plakophilin-2 forward	5′-AAGTAAAGCTGCTTCCGTC-3′
Plakophilin-2 reverse	5′-TTAAACTGAGCCTTCTTGTAGG-3′
Plakophilin-3 forward	5′-ATGTCACAGGGATCCTGTG-3′
Plakophilin-3 reverse	5′-TCAACACCAGGTCTGTGAG-3′
Epiplakin forward	5′-AGCTGGTGAGGATGTATAGAACACAC-3′
Epiplakin reverse	5′-TGTTTGTTGCTGGTTTCCTGC-3′
Nectin-1 forward	5′-CTCAACGTGCAGTATGAGC-3′
Nectin-1 reverse	5′-CTTTGCAGGTGAGCTTCAC-3′

### Whole cell lysates

The cultured cells were washed twice with PBS and lysed on ice in RIPA lysis buffer containing 1 mM phenylmethylsulfonyl fluoride (PMSF, Sigma-Aldrich) for 30 min. Then, the cell lysates were sonicated and centrifuged at 14,000 rpm for 15 min, and the supernatant was collected as a whole cell lysate.

### Subcellular fractionation

The cultured cells were washed twice with PBS and lysed in an appropriate volume of ice-cold cytoplasmic extraction buffer [0.1 mM EGTA, 0.1 mM EDTA, 10 mM HEPES (pH 7.9), and 10 mM KCl] containing 0.5% NP-40, 2 mM DTT and 1 × protease inhibitor. The cell lysates were harvested with a cell scraper, agitated for 20 min at 4 °C, and centrifuged at 14,000 rpm for 3 min. The supernatants were collected as cytoplasmic extracts. For the collection of nuclear extracts, the pellets were resuspended in cytoplasmic extraction buffer and centrifuged at 14,000 rpm for 3 min. Then, the supernatants were discarded, and the pellets were sonicated on ice in nuclear extraction buffer [1 mM EGTA, 1 mM EDTA, 20 mM HEPES (pH 7.9), and 0.4 M NaCl] containing 1 mM DTT and 1 × protease inhibitor. The lysates were centrifuged at 14,000 rpm for 15 min, and the supernatants were collected as the nuclear extracts.

### Western blot analysis

The whole cell lysates or subcellular fractions were boiled with 5 × sodium dodecyl sulfate (SDS) sample buffer for 10 min at 100 °C and subjected to SDS–polyacrylamide gel electrophoresis (SDS-PAGE). After electrophoresis, the proteins were transferred onto polyvinylidene difluoride (PVDF) membranes (Merck Millipore, Billerica, MA, USA). The membranes were incubated in blocking buffer (5% skim milk) for 1 h and incubated overnight at 4 °C with primary antibodies against SIRT1 (1:1000; Cell Signaling Technologies, Beverly, MA, USA), p53 (1:1000; Cell Signaling Technologies), acetyl-p53 (Lys382) (1:1000; Cell Signaling Technologies), vimentin (1:1000; Cell Signaling Technologies), β-actin (1:2000; Cell Signaling Technologies), lamin B (1:4000; ProteinTech), CK-18 (1:2000; ProteinTech), CK-19 (1:2000; ProteinTech), and β-tubulin (1:2000, Sungene Biotech, Tianjin, China). Then, the membranes were washed and incubated at room temperature for 1 h with the appropriate horseradish peroxidase-linked secondary antibodies (1:4000; Abbkine, Redlands, CA, USA). The protein bands were visualized using Pierce™ ECL Western blotting substrate (Merck Millipore).

### Transwell assays

The cell migration and invasion assays were performed using Matrigel-free Transwell chambers and Matrigel-coated Transwell chambers (8.0 μm/pore, Corning, Kennebunk, ME, USA), respectively. Cell suspensions containing 1 × 10^6^ cells/ml were prepared in serum-free medium. A 100-μl volume of the cell suspension was added to the inner side of each chamber, and the outer side was filled with 500 μl of medium supplemented with 10% FBS. After incubation at 37 °C with 5% CO_2_ for 36 h, the cells on the lower surfaces of the Transwell membranes were fixed in 100% methanol, stained with crystal violet, and counted via light microscopy.

### LC–MS/MS-based quantitative proteomic and acetylomic analyses

The cells were collected and lysed to extract the whole cell proteins. Then, the protein extracts were digested with trypsin and labeled with Tandem Mass Tag (TMT, Thermo Fisher Scientific, Waltham, MA, USA). The samples used for the acetylomic analysis were additionally incubated with anti-acetyl lysine antibody-conjugated agarose beads (PTM-104, PTM Biolabs, Hangzhou, Zhejiang, China) to enrich for lysine-acetylated peptides. The LC–MS/MS analyses were performed using an EASY-nLC 1200 System (Thermo Fisher Scientific) coupled to a Q Exactive Plus mass spectrometer (Thermo Fisher Scientific). The resulting MS/MS data were processed by using MaxQuant with an integrated Andromeda search engine (version 1.5.2.8, Max Planck Institute of Biochemistry, Martinsried, Germany). The quantitative method used was TMT-6 plex. The false discovery rate (FDR) threshold was adjusted to < 1%, and the minimum score for the modified peptides was set as > 40.

### Statistical analysis

The statistical analyses were performed using SPSS (version 16.0; SPSS, Chicago, IL, USA). All data are expressed as the mean ± standard deviation (SD). Each error bar indicates the variation among the means of at least three independent experiments. One-way ANOVA was used to perform multiple comparisons of the real-time PCR and Transwell assay data among three or more groups. *Student’s t* test was performed to compare the relative protein levels in the proteomic and acetylomic assays between two groups of cells. The proteomic and acetylomic enrichments were analyzed using Fisher’s exact test. All statistical tests were 2-tailed. A *P* value less than 0.05 was considered statistically significant.

## Results

### Mutation in the NLS sequences prevented SIRT1 from entering the nucleus

**Fig. 1:**
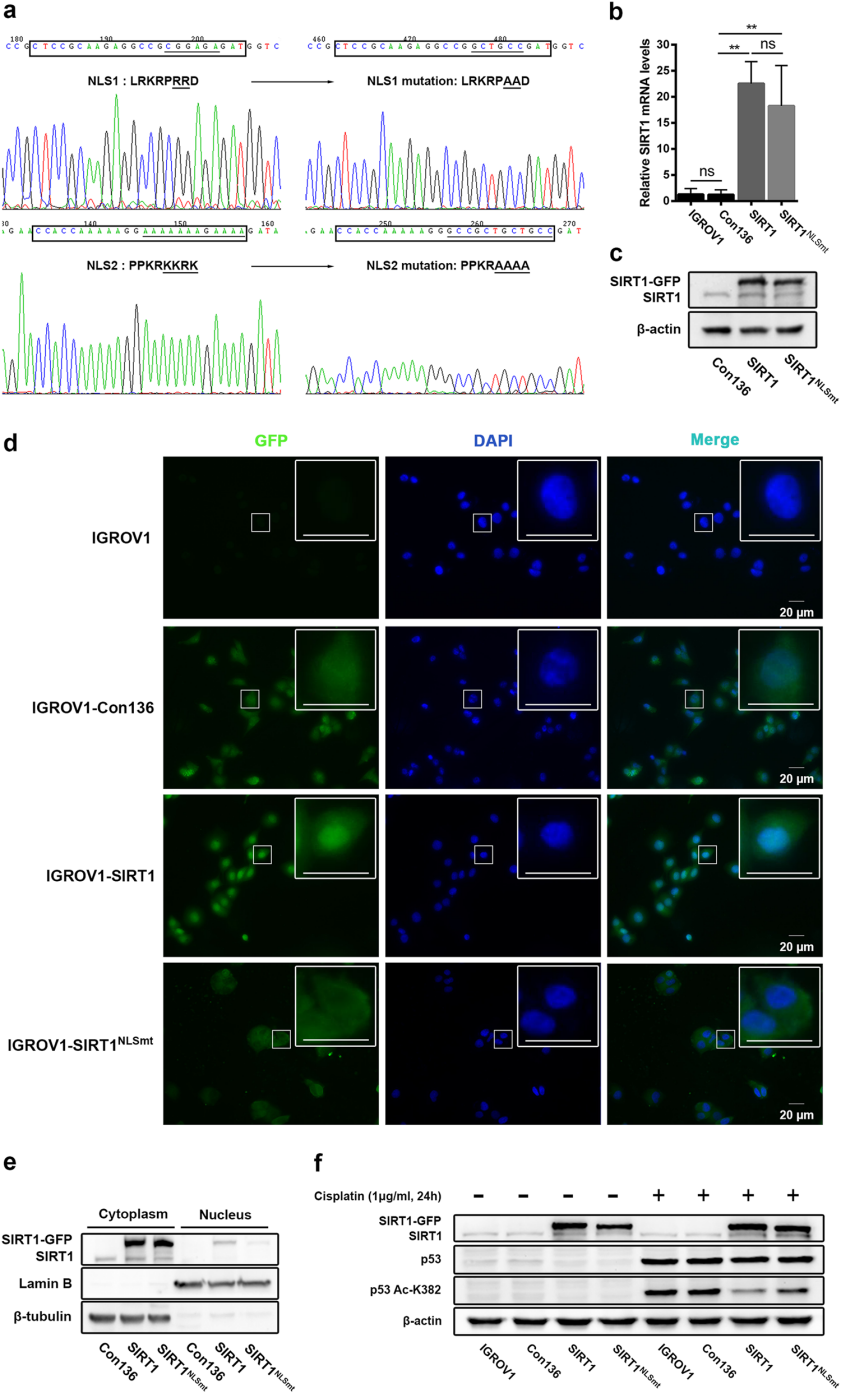
Analyses of SIRT1 subcellular location and expression in ovarian carcinoma IGROV1 cells overexpressing SIRT1 (SIRT1 cells) or NLS-mutated SIRT1 (SIRT1^NLSmt^ cells). **a** The NLS sequence located at amino acids 33–40 (LRKRPRRD) (CTCCGCAAGAGGCCGCGGAGAGAT) was mutated to LRKRPAAD (CTCCGCAAGAGGCCGGCTGCCGAT). In addition, the other NLS sequence located at amino acids 231–238 (PPKRKKRK) (CCACCAAAAAGGAAAAAAAGAAAA) was mutated to PPKRAAAA (CCACCAAAAAGGGCCGCTGCTGCC). **b** SIRT1 mRNA levels as measured by real-time PCR in the parental (IGROV1), Con136, SIRT1 and SIRT1^NLSmt^ cells. Each bar represents the mean ± SD (*n *= 3; **P < 0.01; *ns*, not significant). **c** Western blot analyses of SIRT1 expression in Con136, SIRT1 and SIRT1^NLSmt^ cells. β-Actin was used as a loading control. **d** Immunofluorescence staining of GFP in parental (IGROV1), Con136, SIRT1 and SIRT1^NLSmt^ cells (original magnification: × 400; scale bars: 20 μm). Boxes show the cell details at a higher magnification (scale bars: 20 μm). **e** SIRT1 expression in cytoplasmic and nuclear extracts from Con136, SIRT1 and SIRT1^NLSmt^ cells. β-tubulin and lamin B were used as cytoplasmic and nuclear loading controls, respectively. **f** Protein levels of SIRT1, p53 and acetylated (K382) p53 before and after treatment with 1 μg/ml for 24 h in Con136, SIRT1 and SIRT1^NLSmt^ cells as analyzed by western blotting. β-Actin was used as a loading control

To purposefully study the biological roles of SIRT1 with different subcellular localizations in ovarian carcinoma, IGROV1 cells were stably transfected with lenti-SIRT1 or lenti-SIRT1^NLSmt^. The mutation sites in the two NLS sequences of SIRT1 are shown in Fig. 1a. The overexpressed mRNA and protein levels of exogenous SIRT1 and SIRT1^NLSmt^ were identified by real-time PCR (Fig. 1b) and Western blot analyses (Fig. 1c) of whole-cell lysates, respectively. Because both the lenti-SIRT1-transfected cells (SIRT1 cells) and lenti-SIRT1^NLSmt^-transfected cells (SIRT1^NLSmt^ cells) expressed SIRT1-GFP fusion proteins, green fluorescence was used to determine the subcellular localization of exogenous SIRT1. As shown in Fig. 1d, the green fluorescence signal was observed in the nucleus of SIRT1 cells but was disseminated in the cytoplasm of SIRT1^NLSmt^ cells. Moreover, the SIRT1-GFP protein was almost absent from the nuclear fraction of SIRT1^NLSmt^ cells but enriched in the cytoplasmic fraction (Fig. 1e). These results demonstrate that mutations in the two NLS sequences could prohibit SIRT1 from entering the nucleus.

To verify the deacetylase activity of exogenous SIRT1, we examined the protein levels of p53 and the specific form of p53 acetylated on lysine 382 (Ac-K382), which can be directly deacetylated by SIRT1 [19]. The cisplatin-induced increase in the Ac-K382 p53 protein in both SIRT1 cells and SIRT1^NLSmt^ cells exhibited a prominent decline compared with that in the control vector-transfected cells (Con136 cells, Fig. 1f), further indicating that exogenous SIRT1 possesses deacetylase activity. In addition, we found that the SIRT1^NLSmt^ cells had higher levels of Ac-K382 p53 than the SIRT1 cells. These results further prove that we successfully generated ovarian carcinoma IGROV1 cells overexpressing wild-type SIRT1 or SIRT1^NLSmt^.

### Overexpression of wild-type SIRT1 and SIRT1^NLSmt^ displayed opposite effects on the regulation of cell migration and invasion

**Fig. 2:**
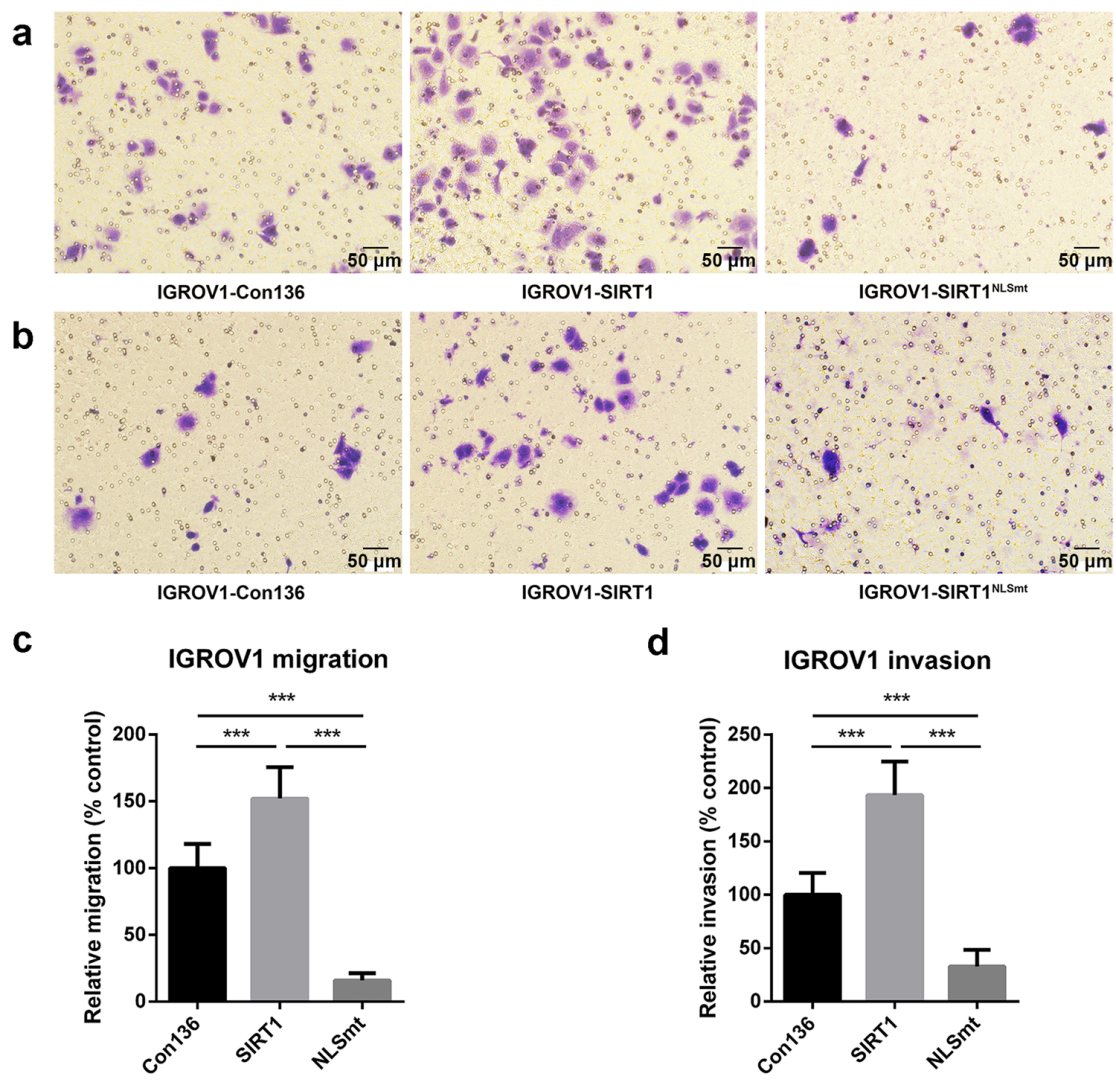
Overexpression of SIRT1 and SIRT1^NLSmt^ had opposite effects on cell migration and invasion. Con136, SIRT1, and SIRT1^NLSmt^ cells passing through the chamber were methanol-fixed, crystal violet-stained, and counted in a × 200 microscopic field. Representative photomicrographs of the cell migration (**a**) and invasion (**b**) assays are shown (scale bars: 50 μm). Relative quantification of migration (**c**) and invasion (**d**) was performed by normalization to the migration and invasion of Con136 cells. The error bars represent the means ± SDs (*n* = 10; ***P < 0.001)

To examine the phenotype of SIRT-1 subcellular localization in ovarian carcinoma cells, Transwell assays were performed to investigate the effect of overexpressed wild-type SIRT1 and SIRT1^NLSmt^ on cell migration and invasion. The results of the migration (Fig. 2a, c) and invasion (Fig. 2b, d) assays show that the overexpression of wild-type SIRT1 significantly increased cell motility, while the overexpression of SIRT1^NLSmt^ markedly decreased cell motility compared with the cell motility of Con136 cells. Given that the parental cells of both the SIRT1 and SIRT1^NLSmt^ cells are the same, our results suggest that this difference in cell motility is most likely related to the SIRT1 subcellular localization.

### Comparative analysis of the proteome and lysine acetylome in SIRT1 and SIRT1^NLSmt^ cells showed differences in the expression and acetylation level of proteins associated with epithelial cell differentiation and cell adhesion

To determine the mechanisms underlying the effects of SIRT1 subcellular localization on migration and invasion in ovarian carcinoma cells, proteomic and acetylomic analyses were performed to systematically identify proteins with different expression or acetylation levels in the SIRT1 cells and SIRT1^NLSmt^ cells.

**Fig. 3:**
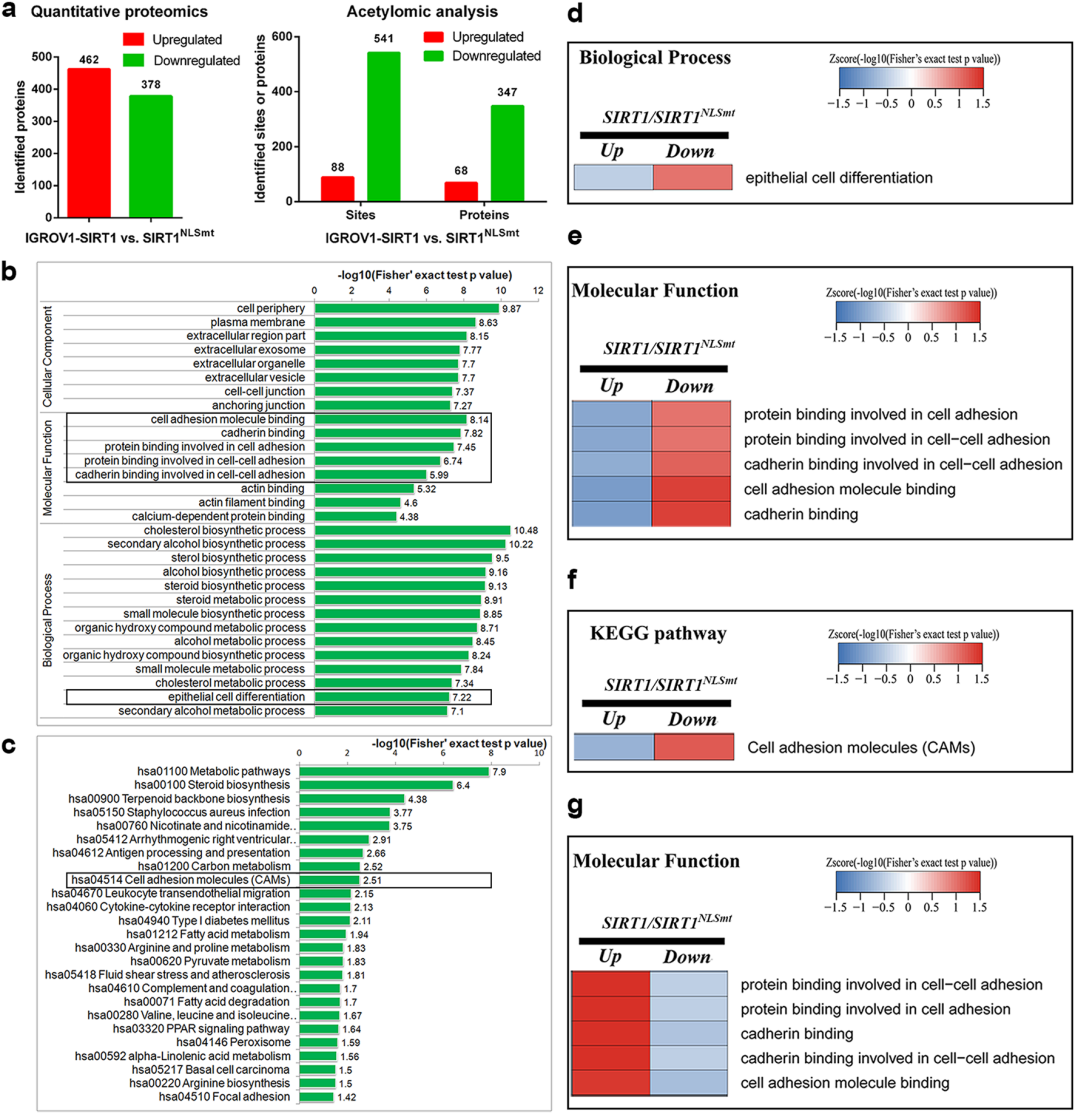
Comparative analysis of the proteome and lysine acetylome in the SIRT1 and SIRT1^NLSmt^ cells. **a** The upregulated and downregulated proteins and acetylated sites in SIRT1 cells compared with those in SIRT1^NLSmt^ cells via quantitative proteomic (left) and acetylomic (right) analyses. **b** Enrichment analysis of the downregulated GO classifications from the proteomic analysis in SIRT1 cells compared with those in SIRT1^NLSmt^ cells. **c** Enrichment analysis of the downregulated KEGG pathways from the proteomic analysis in SIRT1 cells compared with those in SIRT1^NLSmt^ cells. **d–g** Cluster analyses of enriched proteins according to the GO terms and KEGG pathways of proteome and lysine acetylome. Heat maps show the changes in expression levels of epithelial cell differentiation-associated proteins in the GO biological process category (**d**), cell adhesion-associated proteins in the GO molecular function category (**e**) and cell adhesion molecules-associated proteins in KEGG pathways (**f**) from the proteomic analysis, and the changes in acetylation levels of cell adhesion-associated proteins from the acetylomic analysis (**g**)

The proteomic analysis identified 462 upregulated proteins and 378 downregulated proteins in the SIRT1 cells compared with their expression in the SIRT1^NLSmt^ cells (Fig. 3a, left). The acetylomic analysis showed an overwhelming decrease in acetylated proteins and acetylated sites in the SIRT1 cells (Fig. 3a, right), suggesting that the target proteins of SIRT1 are mainly located in the nucleus. To obtain further insight into the biological functions of the identified proteins, the data were further evaluated by performing an enrichment analysis according to the Gene Ontology (GO) classification and Kyoto Encyclopedia of Genes and Genomes (KEGG) pathways. GO terms are divided into the following three broad categories: biological process, cellular component, and molecular function. The most significantly enriched terms in each of the 3 categories are shown in the bar charts (Fig. 3b). The enrichment analysis of the molecular function category indicated that the downregulated cell adhesion-associated proteins were significantly enriched in the SIRT1 cells compared with those in the SIRT1^NLSmt^ cells. In the biological process category, compared with the SIRT1^NLSmt^ cells, we also found a significant enrichment of downregulated epithelial cell differentiation-related proteins in the SIRT1 cells. The KEGG pathway enrichment analysis results revealed a significant decrease in cell adhesion molecule pathway-related proteins in the SIRT1 cells compared with those in the SIRT1^NLSmt^ cells (Fig. 3c). Moreover, the related functional classifications in the different groups were clustered and are displayed as heat maps (Fig. 3d,−g). Consistent with the results of the enrichment analysis, compared with the SIRT1^NLSmt^ cells, in the SIRT1 cells, the proteins associated with epithelial cell differentiation (Fig. 3d) and cell adhesion (Fig. 3e) in the GO categories and proteins participating in cell adhesion molecule KEGG pathways (Fig. 3f) exhibited significant enrichment in the downregulated set rather than in the upregulated set. Interestingly, compared with the SIRT1^NLSmt^ cells, the cell adhesion-associated proteins with an elevated acetylation level in the SIRT1 cells were significantly enriched (Fig. 3g).

Overall, the results of the comparative analyses of the proteome and lysine acetylome indicate that the wild-type SIRT1 protein and cytoplasm-localized SIRT1 protein differentially influence the epithelial phenotype and cell adhesion in ovarian carcinoma cells.

### Overexpression of wild-type SIRT1 and SIRT1^NLSmt^ might have different effects on the epithelial–mesenchymal transition

**Fig. 4:**
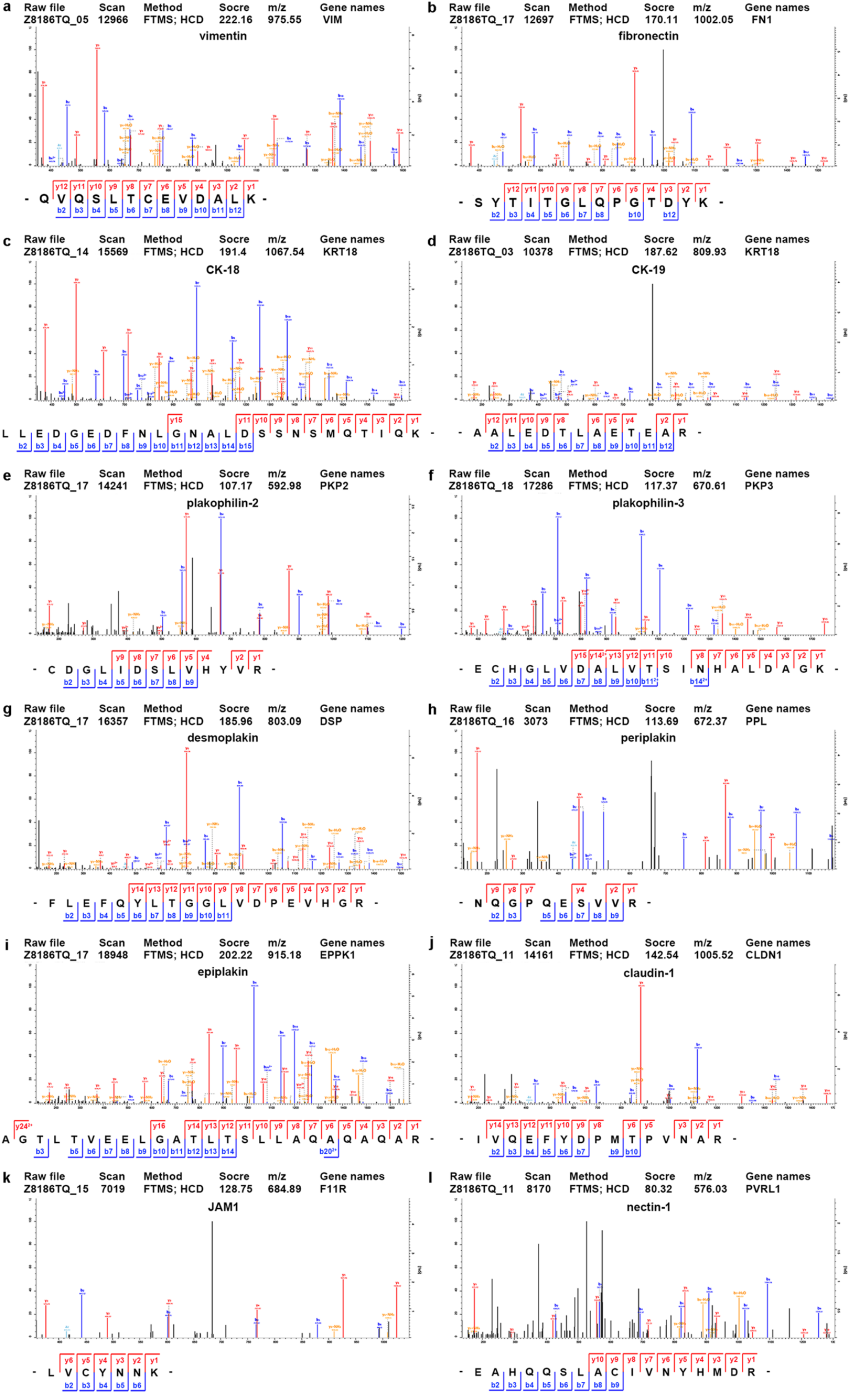
Identification of differentially expressed EMT-associated proteins in the SIRT1 and SIRT1^NLSmt^ cells. MS/MS spectra of specific peptide fragments of vimentin (**a**), fibronectin (**b**), CK-18 (**c**), CK-19 (**d**), plakophilin-2 (**e**), plakophilin-3 (**f**), desmoplakin (**g**), periplakin (**h**), epiplakin (**i**), claudin-1 (**j**), JAM1 (**k**), and nectin-1 (**l**). Blue and red represent the N-terminal fragment ion (B ion) and C-terminal fragment ion (Y ion), respectively. (Color figure online)

**Fig. 5:**
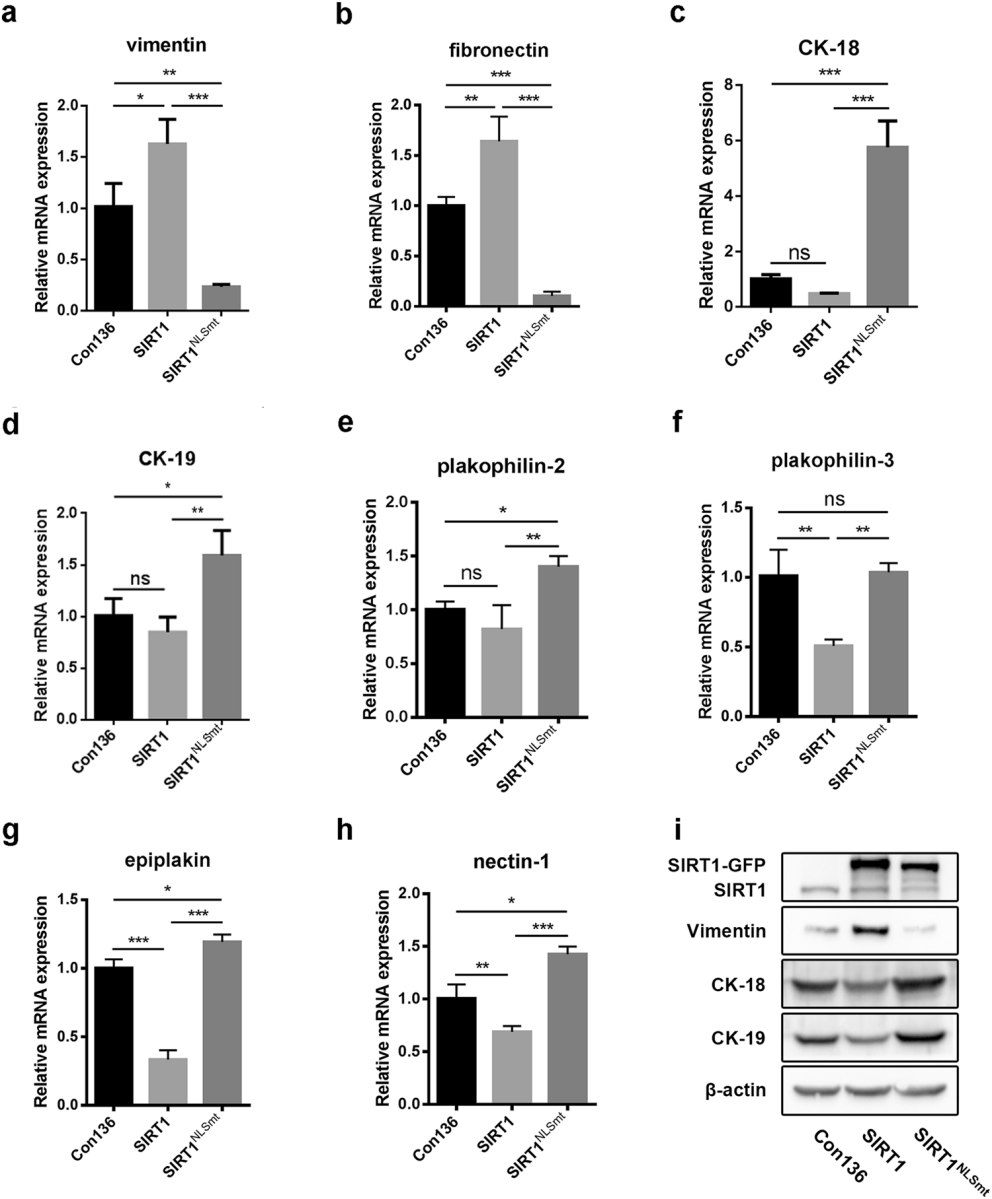
Comparison of the expression levels of EMT-associated proteins in the Con136, SIRT1, and SIRT1^NLSmt^ cells. Relative mRNA levels of vimentin (**a**), fibronectin (**b**), CK-18 (**c**), CK-19 (**d**), plakophilin-2 (**e**), plakophilin-3 (**f**), epiplakin (**g**), and nectin-1 (**h**) as measured by real-time PCR in the Con136, SIRT1, and SIRT1^NLSmt^ cells. Each bar represents the mean ± SD (*n *= 3; ***P < 0.001; **P < 0.01; *P < 0.05; *ns* not significant). **i** Protein levels of vimentin, CK-18 and CK-19 as determined by western blot analyses of the Con136, SIRT1, and SIRT1^NLSmt^ cells. β-Actin was used as a loading control

Given that the epithelial phenotype and cell adhesion are involved in the process of epithelial–mesenchymal transition (EMT), which plays an important role in tumor cell migration and invasion, we focused on EMT-related proteins. The comparison of the protein and acetylation levels of EMT-related biomarkers in the SIRT1 cells and SIRT1^NLSmt^ cells is shown in Table 2. We found markedly increased protein expression levels of the mesenchymal markers vimentin (3.21-fold) and fibronectin (2.27-fold) and significantly reduced protein expression levels of the epithelial markers CK-18 (3.94-fold), CK-19 (8.26-fold), desmoplakin (1.54-fold), plakophilin-2 (1.73-fold), plakophilin-3 (1.60-fold), periplakin (1.56-fold), epiplakin (1.45-fold), claudin-1 (1.86-fold), JAM1 (1.32-fold), and nectin-1 (1.38-fold) in the SIRT1 cells compared with those in the SIRT1^NLSmt^ cells. The MS/MS spectra of peptides from these proteins as determined by the proteomic analysis are presented in Fig. 4. Among these markers, the mRNA levels of vimentin, fibronectin, CK-18, CK-19, plakophilin-2, plakophilin-3, epiplakin, and nectin-1 were confirmed by real-time PCR as shown in Fig. 5a−h. In addition, the protein levels of CK-18, CK-19 and vimentin were further assessed by Western blot analyses (Fig. 5i) and found to be consistent with the proteomic analysis and real-time PCR results.

**Table 2:** Comparison of the protein levels and acetylated sites of EMT-related biomarkers between the SIRT1 and SIRT1^NLSmt^ cells

Proteins	SIRT1/SIRT1^NLSmt^	
	Protein level	Differentially acetylated sites
Vimentin	3.208	K294, K313, K334, K373 and K439
Fibronectin	2.266	ns
CK-18	0.254	K417
CK-19	0.121	ns
Desmoplakin	0.648	K470, K803 and K1590
Plakophilin-2	0.579	ns
Plakophilin-3	0.626	ns
Periplakin	0.642	ns
Epiplakin	0.692	ns
Claudin-1	0.538	ns
JAM1	0.755	ns
Nectin-1	0.724	ns

*EMT* epithelial–mesenchymal transition, *ns* not significant

**Fig. 6:**
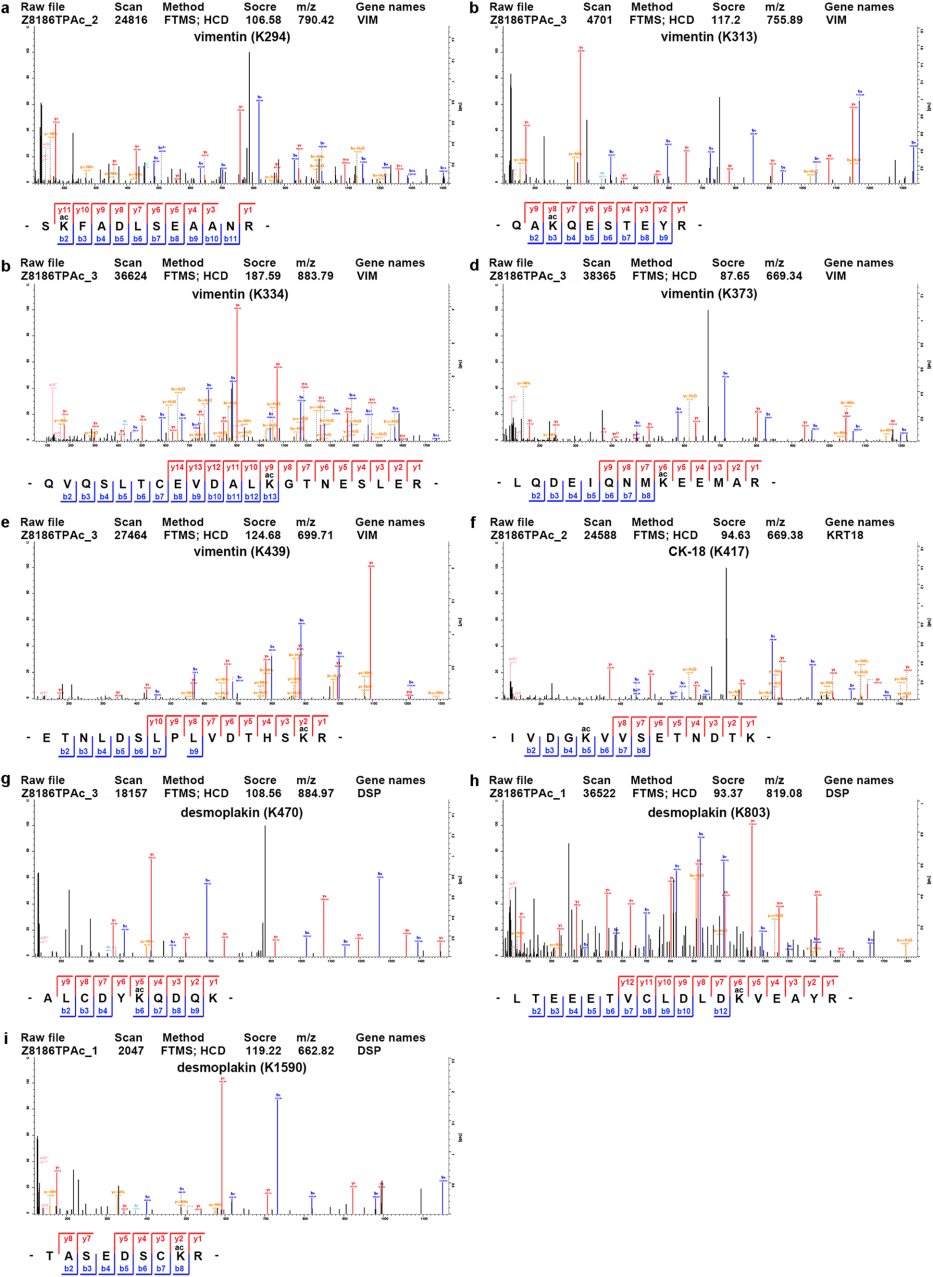
Identification of differentially acetylated lysine residues of EMT-associated proteins in the SIRT1 and SIRT1^NLSmt^ cells. Five MS/MS spectra of vimentin peptide fragments containing acetylated sites at K294 (**a**), K313 (**b**), K334 (**c**), K373 (**d**), and K439 (**e**). One MS/MS spectrum of CK-18 peptide fragment containing acetylated sites at K417 (**f**). Three MS/MS spectra of desmoplakin peptide fragments containing acetylated sites at K470 (**g**), K803 (**h**), and K1590 (**i**)

Notably, among the aforementioned EMT-related proteins, vimentin, CK-18 and desmoplakin were shown to have alterations in the lysine acetylation levels by the acetylomic analysis. Compared with the SIRT1^NLSmt^ cells, the SIRT1 cells exhibited a decrease in vimentin acetylation of K294 (1.91-fold), K334 (2.67-fold), K373 (2.76-fold), and K439 (3.00-fold) and an increase in vimentin acetylation of K313 (1.49-fold). Moreover, in the SIRT1 cells, K417 on CK-18 was detected to have a 2.61-fold hyperacetylation, while desmoplakin showed decreased acetylation of both K470 (1.73-fold) and K1590 (1.88-fold) and increased acetylation of K803 (1.66-fold). The spectra of the peptides containing the aforementioned acetylated lysine residues are shown in Fig. 6.

In conclusion, the above results show that the overexpression of wild-type SIRT1 and SIRT1^NLSmt^ has different effects on the protein and/or lysine acetylation levels of EMT-related biomarkers.

## Discussion

Since 2007, the differential expression of nuclear and cytoplasmic localization has been reported to play multiple roles in cardiomyocyte antioxidation [17, 20], nervous system development [21], neurite outgrowth [22], acute pancreatitis models [23], and osteoarthritis [24]. However, the role of SIRT1 nucleocytoplasmic shuttling in neoplasms has not been addressed.

In addition, in ovarian carcinoma, the roles of SIRT1 in tumor progression remain highly controversial. SIRT1 has been shown to inhibit tumor metastasis in vitro and in vivo by impeding the EMT [25] and reduce cell viability in H_2_O_2_-treated cancer cells by promoting the formation of reactive oxygen species [26], suggesting that SIRT1 plays a role as a tumor suppressor. Conversely, some reports propose that SIRT1 is a tumor promoter. For example, chemotherapy resistance increased the expression of SIRT1 in IGROV1 cells, while SIRT1 silencing led to cell growth arrest and partially enhanced the sensitivity of drug-resistant cells to doxorubicin [27]; the inhibition of SIRT1 resulted in anticancer effects in SKOV3 cells through apoptotic or autophagic cell death pathways [28]; and SIRT1 enhanced cell proliferation, chemoresistance and aggressiveness by upregulating multiple antioxidant pathways to inhibit oxidative stress [29]. However, none of these studies addressed the role of nuclear and cytoplasmic localization of SIRT1.

By creating ovarian carcinoma cell lines overexpressing wild-type SIRT1 and NLS mutated SIRT1 and performing Transwell assays, we found that the overexpression of wild-type SIRT1 significantly increased cell motility, while the overexpression of SIRT1^NLSmt^ markedly decreased cell motility compared with the cell motility observed in the Con136 cells. This result implied that SIRT1 functions as a tumor suppressor or oncogene might depend on its subcellular localization.

EMT has long been thought to be a critical process in tumor progression and metastasis [30]. It is a consecutive process including the dissolution of cell–cell junctions, loss of cell polarity, reorganization of the cytoskeleton, downregulation of the epithelial signature, acquisition of the mesenchymal phenotype and increase in cell motility [31]. The disassembly of epithelial cell–cell contact is considered the first step in EMT [31], and the loss of the expression of E-cadherin and cytokeratins combined with the gain in the expression of mesenchymal biomarkers, such as N-cadherin, vimentin and fibronectin, are the representative characteristics of this process [32]. Epithelial cell contact is maintained through lateral cell–cell junctions (tight junctions, adhesion junctions, gap junctions and desmosomes), which generate apical-basal polarity and interact with the underlying basement membrane through semidesmosomes and α6β4 integrins. In addition, cytokeratins are essential for epithelial cells to stabilize desmosomes and thus contribute to the tissue architecture [33].

By performing unbiased proteomic approaches, we found that SIRT1 might regulate tumor progression via an EMT process. The results of proteomic approaches and Transwell assays demonstrated that the cytoplasmic localization of SIRT1 might suppress the mesenchymal program, activate the epithelial program, and inhibit the migration and invasion of ovarian carcinoma cells, thus providing experimental evidence showing that the role of SIRT1 as a tumor suppressor or oncogene may depend on its subcellular localization as follows: SIRT1 may suppress tumor progression when it is located in the cytoplasm or function as an oncogene when it is located in the nucleus. Therefore, our results may provide an explanation of the highly controversial function of SIRT1 in cancer development by linking its expression to its subcellular localization. Our study also implies that in future clinical applications, not only the protein level but also the subcellular localization of SIRT1 should be considered.

The systematic analyses showed that three EMT-related proteins (CK-18, vimentin and desmoplakin) had remarkable alterations (> 1.3-fold) in both their protein and acetylation levels. For the first time, we found the deacetylation sites on CK-18 (K417), vimentin (K294, K313, K334, K373, and K439) and desmoplakin (K470, K803 and K1590) and identified differences in the acetylation levels of these sites due to differential SIRT1 subcellular localization.

Previous studies have mainly focused on the regulation rather than the function of acetylation in cytokeratin. Therefore, the functions of CK-18, vimentin and desmoplakin acetylation remain unclear. In addition, as a cytoskeleton protein, the acetylation of CK-8 is related to filament stabilization [34]. The deacetylation of CK-8 K207 by SIRT2 results in the increased solubility of CK-8 and reduced filament density surrounding the nucleus [35]. α-tubulin deacetylation by HDAC6 has long been regarded as a critical factor for the destabilization of dynamic microtubules [36]. The hyperacetylation of α-tubulin can stabilize microtubules and decrease cell motility [37, 38]. Thus, we hypothesize that the acetylation status of CK-18 might also be related to filament stabilization and, similarly, that the acetylation levels of vimentin and desmoplakin might be involved in the stability of complexes formed by these proteins. However, the details related to the acetylation of CK-18, vimentin and desmoplakin, such as whether these acetylation changes are directly regulated by SIRT1 and whether they are related to changes in the levels of the associated proteins, need further investigation.

In summary, for the first time, our study showed the possible close relationship between the subcellular location of SIRT1 and the progression of ovarian carcinoma. Cytoplasmic SIRT1 might inhibit the migration and invasion of tumor cells by impeding the EMT. The role of SIRT1 as a tumor suppressor or oncogene may depend on its subcellular localization. Altogether, our findings may highlight a novel function of cytoplasmic SIRT1 in ovarian carcinoma, providing new possible insight for studies investigating the role of SIRT1 in tumor progression.
